# Making periodontal risk visible: the role of artificial intelligence in recall adherence

**DOI:** 10.3389/fdmed.2026.1927437

**Published:** 2026-09-03

**Authors:** Prajakta Karkare

**Affiliations:** General Dental Practice, Bupa Dental, Sydney, NSW, Australia

**Keywords:** artificial intelligence, digital dentistry, patient adherence, periodontal maintenance, periodontal recall, periodontitis, risk communication, supportive periodontal care

## Abstract

Supportive periodontal care is essential for maintaining periodontal stability after active therapy, yet adherence to periodontal maintenance remains difficult. Once bleeding, halitosis or other presenting concerns improve, some patients may interpret the disease as resolved and the maintenance visit as “just a clean.” This Perspective uses the intention–behaviour gap to examine behavioural, financial and health-literacy barriers to continued supportive care. It then considers a narrower, testable hypothesis: artificial intelligence (AI) might assist clinicians in organising clinician-verified periodontal information and presenting it through personalised visual summaries or prompts. Evidence that AI improves periodontal recall attendance is currently sparse; most dental AI research concerns image interpretation or classification, and the only directly relevant periodontal adherence study is proof-of-concept. Proposed risk projections are therefore presented as a distant research concept rather than a clinically validated tool. AI should not replace clinical judgement, and any future system would require validation, transparent uncertainty, consent, governance, equity safeguards and comparison with simpler non-AI interventions.

## Introduction

A persistent challenge in periodontal care is maintaining engagement after active therapy. Patients may initially accept treatment because bleeding, halitosis, mobility or fear of tooth loss makes the problem visible and urgent. When these concerns improve, the perceived threat may fall even though susceptibility to recurrence remains. The maintenance visit may then be reframed as “just another clean” rather than continued disease control. Supportive periodontal care is associated with better long-term outcomes, but attendance remains variable (1–5).

The intention–behaviour gap offers a useful explanation. A patient may understand and agree with a recommended interval in the clinic, yet later postpone the visit because of work, family responsibilities, health problems, cost or competing priorities. Knowledge and intention are therefore necessary but not always sufficient for action. This is particularly relevant to periodontitis, which may progress with few symptoms and whose clinical measures—probing depth, attachment loss, bleeding on probing and radiographic bone loss—can be difficult for patients to translate into personal risk (6–11).

Financial and cognitive burdens may reinforce disengagement. Periodontal maintenance requires repeated expenditure for a benefit that is largely preventive and future-focused. At the same time, patients may be asked to interpret several interacting factors, including plaque control, smoking, diabetes, residual pocketing, furcation involvement and attendance history. A brief verbal explanation may not leave a clear understanding of what has improved, what remains unstable and why the recommended interval matters. Older patients managing multiple conditions and younger patients balancing work or family commitments may also experience appointment fatigue, although “recall fatigue” is proposed here as a clinical description rather than an established periodontal construct (5, 7–11).

These behavioural, financial and cognitive barriers suggest that the challenge is not simply one of patient knowledge. Many patients are informed about their diagnosis, understand the recommendation at the time of consultation and may even intend to return. However, that intention can weaken once symptoms improve and competing priorities re-emerge. The difficulty therefore lies not only in providing information, but in helping patients retain, interpret and act upon that information over time.

Traditional approaches include verbal explanation, written instructions, recall scheduling and reinforcement during maintenance appointments. More recently, digital reminders, mHealth applications and periodontitis-focused digital companions have been introduced to support ongoing communication and self-management (12, 13). These approaches provide an important foundation and some have demonstrated improvements in patient engagement and oral-hygiene behaviours. However, evidence for sustained periodontal maintenance attendance remains limited.

Despite these developments, many patients continue to disengage from supportive periodontal care. Existing systems often provide reminders or educational information but may not fully address how patients interpret risk. Periodontal information remains difficult for many patients to visualise, particularly when disease progression is gradual, asymptomatic and influenced by multiple interacting factors.

It is within this context that artificial intelligence becomes relevant; artificial intelligence is discussed in this Perspective as a potential communication adjunct rather than a clinical decision-making tool. Current evidence is insufficient to support claims that AI improves periodontal recall attendance. Indeed, the only directly relevant periodontal study identified to date is a proof-of-concept investigation.

Proof-of-concept studies are designed to establish technical and clinical feasibility rather than demonstrate effectiveness. In the study by Miyamoto et al., an AI-supported adherence platform was developed and evaluated as an early-stage feasibility concept, but long-term improvements in maintenance attendance, behavioural adherence and periodontal outcomes remain unproven. Consequently, the study should be interpreted as preliminary evidence supporting further investigation rather than confirmation of clinical benefit (14).

The potential additional contribution of AI would be its capacity to combine several clinician-verified inputs and tailor the presentation to an individual patient. For example, a system might organise changes in bleeding scores, plaque scores, probing depths, radiographic bone loss, medical risk factors and attendance history into a concise summary. This could help a clinician explain why a patient who feels better may still require supportive care. However, personalisation does not necessarily improve behaviour, and the same summary could often be generated through conventional digital records. The incremental value of AI remains an empirical question and requires prospective evaluation (12–18).

**Figure 1:**
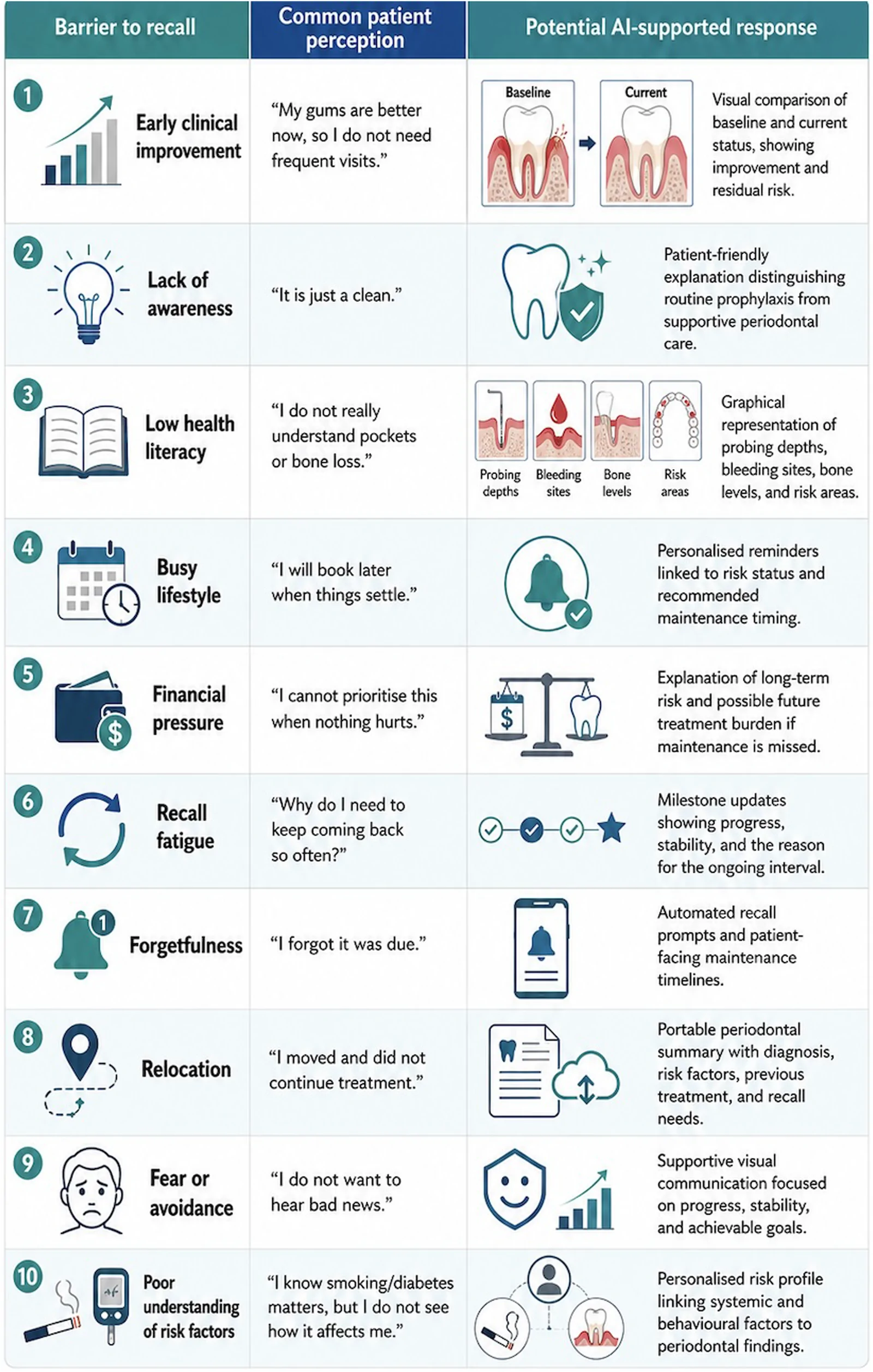
Pictorial representation of potential AI-Supported Responses to address periodontal recall barriers.

Figure 1 maps common barriers, patient perceptions and possible AI-supported responses. Its purpose is to provide a framework for research and communication, not to imply that the proposed responses are effective. Patient-facing summaries could show baseline findings, clinician-verified improvement and persistent high-risk sites. Their effects on understanding, anxiety, trust, attendance and clinical outcomes would need prospective evaluation.

A more distant possibility is scenario-based risk communication. Rather than predicting that a particular patient will lose a specified amount of periodontal support over 10 or 20 years, a future system might present conditional ranges: what may occur if the current risk profile persists, if supportive care and risk-factor control are maintained, or if attendance becomes irregular. Even this approach is currently unsupported for patient-facing periodontal use. Individual progression is biologically and behaviourally heterogeneous, and available models do not justify reliable long-term personal forecasts. Development would require large longitudinal datasets, external validation, calibration across populations, transparent confidence intervals and clinician verification. Until these requirements are met, such outputs should be treated as a research hypothesis, not a clinical promise (6, 14–18).

AI-supported risk communication also creates potential harms. A projection may falsely reassure a patient, produce unnecessary alarm or feel coercive when linked to treatment acceptance. Incomplete charting, inconsistent radiographs or inaccurate medical histories could generate misleading outputs. Clinicians would need to know how the model was trained, for which populations it was validated, when it should not be used and who is accountable when an output is wrong (17–20).

Data governance cannot be treated as an afterthought. Combining periodontal charts, radiographs, medical conditions and behavioural information raises questions of informed consent, data minimisation, secondary use, cybersecurity, ownership, auditability and cross-jurisdictional regulation. Bias is also a concern: models developed from unrepresentative populations may perform unevenly, while patients with limited digital access, language barriers or low digital literacy may be disadvantaged. Human oversight must remain explicit, with the treating clinician responsible for diagnosis, interpretation and recall recommendations (17–20).

Another practical question is whether additional digital contact would change behaviour or simply become another ignored notification. Reminder fatigue, novelty effects and variable patient preferences should be studied. A useful intervention may require more than frequency or visual sophistication; it may need timely delivery, clear action, patient choice and integration into the clinical relationship. Comparative studies should therefore test AI-supported communication against standard clinician explanation, automated reminders and non-AI personalised summaries (8, 9, 12–14).

A structured, portable periodontal summary may still be valuable for continuity of care when patients move between practices or countries. It could include diagnosis, treatment completed, residual concerns, risk factors, radiographic findings and the recommended maintenance interval. This function does not inherently require AI. AI might assist with extracting or translating information, but interoperability, accuracy and governance would need to be established before routine use (13, 16–20).

In general practice, I had limited exposure to Pearl AI radiographic software during a trial and formed the personal impression that some patients appeared more engaged when findings were displayed with visual overlays. This was not systematically measured and may reflect novelty, clinician enthusiasm or expectation bias. It should not be interpreted as evidence of improved treatment acceptance or recall adherence. The observation only prompted the research question of whether visual presentation merits formal evaluation (15–18).

Future research should begin with modest, measurable questions. Does a clinician-verified visual summary improve understanding of supportive periodontal care? Does it alter anxiety, trust or intention to return? Does AI tailoring add benefit beyond the same information presented through a conventional digital template? Subsequent studies could evaluate attendance, periodontal stability, cost-effectiveness, equity and whether any effect persists after the novelty of the technology diminishes. Research reporting should clearly describe the data used, external validation, model limitations, human oversight and adverse effects (12–20).

For supportive periodontal care to be effective, patients must understand it as continuing disease control rather than optional cleaning. The intention–behaviour gap helps explain why agreement in the clinic may not translate into attendance. AI-supported communication is a plausible topic for investigation, but it is not an established solution. Most proposed functions overlap with existing digital tools, and scenario-based projections remain especially uncertain. The appropriate next step is comparative, ethically governed research that tests whether AI adds meaningful value to simpler communication and reminder strategies.

## Data Availability

The original contributions presented in the study are included in the article/Supplementary Material, further inquiries can be directed to the corresponding author.
